# Impact of 6-Month Aerobic Exercise on Alzheimer's Symptoms

**DOI:** 10.1177/0733464813512895

**Published:** 2013-12-11

**Authors:** Fang Yu, William Thomas, Nathaniel W. Nelson, Ulf G. Bronas, Maurice Dysken, Jean F. Wyman

**Affiliations:** 1 University of Minnesota, Minneapolis, USA; 2 University of St. Thomas, Minneapolis, MN, USA

**Keywords:** exercise, Alzheimer's disease, dementia, aging, cognition

## Abstract

Little is known about how aerobic exercise affects Alzheimer's disease (AD). The purpose of this pilot study was to test the impact of 6-month cycling on AD symptoms in community-dwelling older adults with mild-to-moderate AD, using a single-group, repeated-measures design (*n* = 26). AD symptoms were measured with the AD Assessment Scale–Cognitive (ADAS-Cog), Disability in AD (DAD), and Neuropsychiatric Inventory–Caregiver (NPI-Q) scales at baseline, 3 and 6 months. Data were analyzed using mixed linear models. The ADAS-Cog, DAD, and NPI-Q severity scores remained unchanged over the 6-month period, while caregiver distress decreased 40% (p < .05). We conclude that aerobic exercise may reduce AD symptoms and appears effective in decreasing caregiver distress. Further randomized controlled trials are needed to examine the effects of aerobic exercise in AD.

## Background

Alzheimer's disease (AD) affects 5.2 million Americans at present and will inflict 14 million by 2050 if no cure is found (Alzheimer's Association, 2013). The cost of AD to society is substantial (Murman et al., 2002; O'Brien, Shompe, & Caro, 2000; Taylor, Schenkman, Zhou, & Sloan, 2001), estimated at $203 billion in 2013 (Alzheimer's Association, 2013). Moreover, AD dramatically affects family caregivers who live and care for 70% of the AD patients. In 2012, 15.4 million American caregivers provided 17.5 billion hr of unpaid care, which amounted to $216 billion; they also experienced new diseases and exacerbation of existing diseases due to caregiving, which reached another $9.1 billion (Alzheimer's Association, 2013).

The core symptoms of AD include cognitive impairment in at least two cognitive domains, decline in the ability to perform activities of daily living (ADLs), and manifestation of behavioral and psychological symptoms of dementia (BPSD), which are collectively known as the AD triad (American Psychiatric Association [APA], 2000). Cognitive impairment contributes to ADL decline and BPSD in AD (Yu, Kolanowski, Strumpf, & Eslinger, 2006); hence, cognition has been a major outcome investigated in AD randomized controlled trials (RCTs). Currently, four drugs have been approved by the Federal Drug Administration for treating cognitive impairment in AD, including three cholinesterase inhibitors (donepezil, galantamine, and rivastigmine) and one NMDA receptor antagonist (Memantine). A recent meta-analysis of 10 AD drug RCTs showed that the AD Assessment Scale–Cognitive Subscale (ADAS-Cog) scores decreased (indicating improvement) in the cholinesterase inhibitor treatment group but increased (indicating worsening cognition) in the control group. However, the mean difference in the ADAS-Cog scores between the two groups was −2.37 (95% CI [−2.73, −2.02]; range = −1.10 to −3.90), suggesting that pharmacotherapy has, at best, a modest effect on stemming cognitive decline in AD (Birks, 2009). The natural course of the ADAS-Cog scores is an increase of 2.4 to 3.9 points over 6 months in AD (Doraiswamy, Kaiser, Bieber, & Garman, 2001). The modest effects of AD drugs, along with their potential side effects, increased mortality from drug treatment for BPSD (Birks, 2009; Setoguchi et al., 2008), and the cost of drug treatment, especially among the uninsured (Bond et al., 2012; Pouryamout, Dams, Wasem, Dodel, & Neumann, 2012), suggests that alternate approaches to the treatment of AD is much needed.

Aerobic exercise represents one example of a behavioral intervention that may have merit in treating AD. Aerobic exercise could be potentially effective because evidence suggests that it may improve cognition through biologically sound pathways such as increasing neurogenesis and synaptogenensis and reducing β-amyloid accumulation in the brain (Cotman & Berchtold, 2007). Epidemiological studies show that midlife exercise was associated with delayed AD onset (Andel et al., 2008; Cotman & Berchtold, 2007; Fratiglioni, Paillard-Borg, & Winblad, 2004), whereas RCTs indicate that aerobic exercise improved cognition in adults without AD and those with cognitive impairment and dementia (Colcombe & Kramer, 2003; Etnier, Nowell, Landers, & Sibley, 2006; Heyn, Abreu, & Ottenbacher, 2004; Smith et al., 2010). However, few studies have examined the application of aerobic exercise in AD samples. Emerging studies did not use the ADAS-Cog, but they did show that aerobic exercise increased cognition as measured by the Mini-Mental State Examination (MMSE) scores by 2.3 to 3.5 points (*p* < .001, *n* = 35; Palleschi et al., 1996), reduced ADL disability by 6.7% (Rolland et al., 2007), and decreased BPSD as measured by the Neuropsychiatric Inventory–Caregiver (NPI-Q) scores from 43 to 35.7 (*p* < .05, *n* = 23) in older adults with AD (Landi, Russo, & Bernabei, 2004). While the above findings indicate that aerobic exercise might indeed be a potential intervention for AD, few studies have evaluated the efficacy of a well-designed and delivered aerobic exercise intervention in a clearly characterized AD sample (Forbes et al., 2008; Yu, 2011). With few exceptions (Cyarto et al., 2010), the ADAS-Cog has not been used in exercise studies in AD.

Hence, it is critically important to evaluate the efficacy of aerobic exercise interventions on AD symptoms using standard measures so that the effect sizes across different interventions (i.e., drugs vs. exercise) and various studies can be compared. The current study examined the impact of a 6-month standardized, moderate intensity cycling intervention on cognition, function, BPSD, and caregiver distress in community-dwelling older adults with mild-to-moderate AD. Based on the natural course of 2.4 to 3.9-point increase in the ADAS-Cog over 6 months in AD (Doraiswamy et al., 2001), our priori hypothesis was that the cycling intervention would maintain cognition. We further hypothesized that participants will be able to maintain their ADLs and reduce BPSD severity and caregiver distress over 6 months.

## Method

### Design

This study used a single-group repeated-measures design to deliver a 6-month cycling intervention to community-dwelling older adults with mild-to-moderate AD. The intervention was a standardized, supervised, and individualized moderate intensity cycling for 15 to 45 min a session (excluding 10-min warm-up and 10-min cool-down activities), 3 times a week for 6 months. Selected outcomes included cognition, ADLs, BPSD, and caregiver distress, which were measured at baseline, 3 months, and 6 months, respectively, by trained research assistants (RAs) who were blinded to the study aims and baseline scores. The study was approved by the University of Minnesota Institutional Review Board (IRB).

### Sample

The study sample included community-dwelling older adults with mild-to-moderate AD and their family caregivers. Inclusion criteria for older adults with mild-to-moderate AD were as follows: (a) 60 years of age or older; (b) living in the home, independent apartment, assisted living, or other community setting other than nursing home; (c) diagnosis of probable AD as verified by a medical provider; (d) MMSE score between 12 and 24, which is the established range for defining mild-to-moderate AD (Folstein, Folstein, & McHugh, 1975); (e) CDR score of 1 to 3, which is the established range for defining mild-to-moderate AD (Morris, 1994); (f) resting heart rate less than 100 beats per minute to ensure exercise safety; (g) receipt of medical clearance for the cycling intervention from the primary care provider and from a cardiologist for those with known cardiac history; and (h) English as a primary language. Older adults with mild-to-moderate AD were excluded if they had contraindications for aerobic exercise (i.e., severe heart block), unstable medical conditions (i.e., uncontrolled hypertension, hip fracture, or deep vein thrombosis) in the past 6 months, and new symptoms that had not been evaluated by a health care provider (i.e., severe shortness of breath). Older adults with mild-to-moderate AD were further excluded if they had other neurological diseases such as stroke or movement disorder; psychiatric disorders such as schizophrenia, alcohol, or chemical dependency; or significant depressive symptoms (Geriatric Depression Scale Short Form score > 5) that might independently contribute to cognitive impairment and complicate the interpretation of cognitive changes from the intervention in the past 5 years (Burke, Roccaforte, & Wengel, 1991).

Inclusion criteria for family caregivers were as follows: (a) self-identified as the primary care provider for the older adult with mild-to-moderate AD and (b) their care-recipients had been qualified for enrollment. All the 26 family caregivers gave informed consent to participate in the study themselves.

### Setting

In-person interviews took place at the university's Clinical and Translational Science Institute (CTSI). The intervention was delivered at two sites: a YMCA gymnasium and a senior facility, whichever was more proximate to the older adult with mild-to-moderate AD's residence to minimize travel time. The procedures for emergency at all three sites were to call 911. All three sites were in close proximity to tertiary hospitals and located in a Midwestern city.

### Outcome Variables and Measurements

The outcome variables in this study included cognition, ADLs, BPSD, and caregiver distress. Cognition was defined as information processing of one's psychological function and was measured using the ADAS-Cog, which assesses orientation, memory, recall, language, and praxis with a total score of 0 to 70 (higher scores = worse cognition). The ADAS-Cog has an interrater reliability of .65 to .99 and test–retest reliability of .51 to 1 (Rosen, Mohs, & Davis, 1984).

ADLs were defined as the ability to perform basic and instrumental ADLs and were measured by the Disability Assessment for Dementia (DAD). This measure assesses 17 basic and 23 instrumental ADL items based on family caregiver reports with a total score of 0 to 40 (higher scores = greater disability). The DAD demonstrated no gender bias with .95 test–retest and .96 inter-rater reliability (Gelinas, Gauthier, McIntyre, & Gauthier, 1999).

BPSD was defined as symptoms of disturbed perception and thoughts, mood, affect, and behaviors associated with AD. Caregiver distress referred to the emotional and physical strain from caring for a family member with AD. BPSD and caregiver distress were measured by the NPI-Q, which asked the family caregivers to rate the presence of 12 BPSD symptoms in the past month as “yes” or “no” and, then, the severity of present symptoms from 1 to 3. The BPSD severity score ranges 0 to 36 with an interscale correlation of .91 for severity rating, and higher scores indicate more BPSD severity (Cummings, 1997). The NPI-Q further asked the caregivers to rate the impacts of BPSD on caregiver distress from 1 to 5. The caregiver distress subscale score ranges 0 to 60 with an interscale correlation of .92 and higher scores indicate greater caregiver distress (Cummings, 1997).

### Study Procedure

#### Staff training

All study staff completed a 4-hr didactic training about understanding AD, communications with older adults with AD, and the study protocol by the first author. Data collectors were blinded to all previous scores. Staff then observed the first author conduct all aspects of the study and then performed different tasks of the study under the first author's supervision through 1-week practice training. Staff began to work independently once they reached 100% agreement with the first author about the protocol implementation. The staff was re-trained during regular weekly staff meetings and if deviations from the protocol were observed. The first author randomly checked 10% of the exercise sessions and educated the staff if deviations from the protocol were observed to ensure treatment fidelity.

#### Enrollment qualification

Community-dwelling older adults with mild-to-moderate AD were screened via three steps:

1. Phone screen by an undergraduate RA with the family caregiver and the older adult with mild-to-moderate AD (30 min). The focus of the phone screen was to elicit the present and past health and exercise history, AD diagnosis, and medications;
2. In-person interview of potential older adult with mild-to-moderate AD who passed the phone screen. The RA and the first author obtained informed consent from the older adults with AD and family members. Informed consent was also obtained from the family caregivers for their own participation in the study. After the study was explained in detail, the RA administered a 10-item questionnaire about the study to the older adult with mild-to-moderate AD. If he or she failed to answer 80% of the questions correctly, the study was re-explained and the questionnaire was re-administered. If the older adult with mild-to-moderate AD still could not answer 80% or more correctly, then the family member in the descending order of spouse, adult child, sibling, and relatives gave surrogate consent on behalf of the older adult with mild-to-moderate AD, while the older adult with mild-to-moderate AD gave assent. The involvement for the family caregiver was also explained in detail. The whole consent process took about 35 min. Then the PI administered the CDR to the family caregiver, while the RA gave the MMSE to the older adult with mild-to-moderate AD in a different room. At the end of the interview with the family caregiver, the first author proceeded to interview the older adult with mild-to-moderate AD using the CDR while remained blinded to the MMSE score. At the end of the interview, the first author scored the CDR and conducted a brief physical examination with the older adult with mild-to-moderate AD, while the RA scored the MMSE independently. Afterward, the first author and the RA discussed the next step with the older adult with mild-to-moderate AD and the family members: proceeding to Step 3 or informing them that they did not meet the enrollment criteria;
3. Medical clearance and AD diagnosis verification (1-2 weeks). If an older adult with mild-to-moderate AD had an MMSE score between 12 and 24 and a CDR score between 1 and 3, that is, meeting the definition for mild-to-moderate AD, a letter was faxed to the older adult with mild-to-moderate AD's primary care provider and health care provider who diagnosed AD to obtain medical clearance for the older adult with mild-to-moderate AD to participate in the study and verify AD diagnosis. A medical clearance letter was sent to the potential older adult with mild-to-moderate AD's cardiologist if he or she had a cardiac history. Older adults with verified AD diagnosis and exercise safety were enrolled in the study. Their family caregivers were also enrolled in the study.

#### Data collection

Once qualified, older adults with mild-to-moderate AD underwent baseline data collection at the CTSI. A trained undergraduate RA who was blinded to the study aims administered the cognitive instruments to the older adult with mild-to-moderate AD and collected data on ADLs and BPSD from the family caregiver. The exercise interventionist, an occupational therapist with 20 years of experience at cardiac rehabilitation, administered the 6-min walk test (Ries, Echternach, Nof, & Blodgett, 2009) and the shuttle walk test on a different day (Singh, Morgan, Hardman, Rowe, & Bardsley, 1994). Those two tests were used to estimate the heart rate reserve for each older adult with mild-to-moderate AD and guide cycling prescription.

#### Cycling prescription and delivery

Moderate intensity was primarily prescribed as 65% to 75% of the heart rate reserve, which was defined as the difference between the resting heart rate post 10-min resting and the highest heart rate recorded from the baseline shuttle walk test and 6-min walk test. For older adults with AD with irregular heart rhythm or taking heart rate-altering drugs such as beta-adrenergic blocking agents, moderate intensity was prescribed as 12 to 14 on the 6 to 20 Borg Rating of Perceived Exertion (RPE) scale (Borg, 1982). A previous study has shown that older adults with mild-to-moderate AD could improve their aerobic capacity from participating in 6-month cycling that was prescribed based on a RPE scale (Yu, Savik, Wyman, & Bronas, 2011).

Within a week of completing the baseline data collection, the older adults with mild-to-moderate AD began the 6-month moderate intensity cycling intervention on Precor™ recumbent stationary cycles (Woodinville, WA) or LIVESTRONG® R1× recumbent bike (Cottage Grove, WI), 3 times a week. Use of different cycles was justified because cycling prescriptions were individualized based on heart rate reserves and RPE. The duration of cycling at moderate intensity was progressively increased by 5-min increments from 15 min initially to 45 min per session. The exercise interventionist supervised two to three older adults with mild-to-moderate AD a session, whereas an undergraduate RA assisted the exercise interventionist in each session and provided transportation to the older adults with mild-to-moderate AD using a rented university vehicle.

In each session, older adults with mild-to-moderate AD wore the Polar™ F7 heart rate monitor (Polar Electro Finland Oy, Kempele, Finland) and completed 5-min range of motion activities, 5-min cardiac warm-up (marching in place and low intensity cycling), 15- to 45-min cycling at moderate intensity, 5-min cardiac cool-down cycling, and 5-min stretches. The cycling dose was started at 40% to 55% of the heart rate reserve (or RPE 9) for 15 min initially and was increased by 5-min increments after they completed a prescribed dose 3 times in a row. Once older adults with mild-to-moderate AD could cycle for 30 min at 40% to 55% of the heart rate reserve (or RPE 9), the cycling intensity and duration were alternatively increased by 10% of the heart rate reserve (or RPE 1) or 5 min, respectively, until older adults with mild-to-moderate AD could cycle for 45 min at 65% to 75% of the heart rate reserve or RPE 12 to 13 (Yu, 2013). The heart rate and RPE were assessed in the last 15 s of each 5-min interval, and the proper use of the RPE was continuously reinforced. The exercise interventionists further monitored blood pressure every 10 to 15 min, administered the talk test (older adult with mild-to-moderate AD's ability to talk a sentence without losing breath), and over-exertion signs and symptoms. The PI randomly checked 10% of the sessions each month to ensure treatment fidelity.

### Analysis

Descriptive statistics were reported for baseline characteristics. Longitudinal changes in outcomes from baseline to 3 and 6 months were modeled with a mixed-effects linear model with study month as fixed effect and a random intercept for each older adult to account for the correlation between measurements from the same person. Baseline characteristics were controlled for as covariates. Mixed-effect models accommodate missing data, so no imputation was used for missing data. All analyses were performed using SAS Version 9.3 (SAS Institute, Inc. 2010, Cary NC).

## Results

Older adult participants were non-Hispanic Whites, aged 60 to 91 years. About 75% were overweight, scored 19 or higher on the MMSE, and had CDR score of 1 (see Table 1). Family caregivers were on average 14 years younger than the older adults with mild-to-moderate AD but ranged from 38 years younger to 4 years older. Of the 28 participants, 26 (92%) completed the 3-month data collection and 22 (79%) completed the 6-month data collection. Reasons for dropping out were relocation (*n* = 2), study-unrelated fractures (*n* = 2), study-unrelated transient ischemic attack (*n* = 1), and busy schedule (*n* = 1). We report results for the 26 participants who completed 3-month data collection and their 26 family caregivers.

Adherence to the exercise program was high. Participants attended 83% of their prescribed sessions (65 sessions on average with a range of 30 to 72, and *SD* of 13.2), and 85% of the participants attended 70% or more of the 72 sessions. Except for one outlier who achieved the cycling prescription only in 4% of the sessions, the remaining 25 participants (96%) achieved the session cycling prescription on an average 90% of their sessions (range = 73%-100%).

Participants maintained their mean ADAS-Cog, DAD, and NPI-Q severity scores, with no significant changes from baseline (see Table 2). Caregiver distress, as measured by NPI-Q distress, decreased significantly by almost 40% from baseline (see Table 2). Baseline characteristics such as baseline ADAS-Cog score, MMSE score, CDR score, years of age, and body mass index (BMI) did not appear to show associations with outcome changes. There were no differences in ADAS-Cog, DAD, and NPI-Q severity scores from 3 to 6 months; however, caregiver distress significantly decreased from baseline by 8% at 3 months and 32% from 3 to 6 months (*p* < .05).

## Discussion

In this study, community-dwelling older adults with mild-moderate AD maintained their cognition over the 6-month period while they participated in a supervised, standardized, and moderate intensity cycling intervention, and this program significantly reduced distress in primary family caregivers. Our cognitive finding is consistent with the results from other aerobic exercise studies (Arkin, 2003; Kemoun et al., 2010; Rolland et al., 2007; Williams & Tappen, 2007; Williams & Tappen, 2008). Adherence in this study was toward the high end of the reported 17% to 90% adherence; however, it is often unclear how the adherence was determined in those studies that used low exercise doses and lacked clear description of the prescribed exercise doses (Yu, 2011).

The implications of our finding are significant because cognitive decline, not cognitive stabilization, marks AD trajectory. Based on pooled data from AD drug RCTs, the mean trajectory for AD cognitive decline is an average 2.4-to 3.9-point increase in the ADAS-Cog scores over 6 months, indicating worsening cognition (Doraiswamy et al., 2001). The participants' ADAS-Cog scores increased by only 1.9 points in this study. Moreover, participants showed no increase in the DAD scale and the NPI-Q severity over the 6-month period. This finding is also consistent with other studies that showed that exercise reduced ADL declines and BPSD severity in nursing home residents with AD (Landi et al., 2004; Rolland et al., 2007).

Our finding further showed a significant reduction in caregiver distress by 40%. It is well established that a 30% reduction in baseline score of the NPI-Q is clinically meaningful. This finding is consistent with what caregivers reported during the focus group interviews at the end of the study that the exercise program “was socially rewarding,” “increased physical strength,” “was a positive experience,” “led to improved attitude in older adults with AD,” and “reduced caregiver stress” (Yu & Swartwood, 2012). This finding was also expected because the current literature supports the idea that respite reduces caregiver stress, and our study was designed as a respite for family caregivers. Our RAs picked up the older adult participants from their residence, drove them to the exercise site to engage in cycling, and transported them back home after the exercise sessions; hence, our study provided care-givers about 8- to 10-hr respite per week, which caregivers repeatedly acknowledged during the study. However, findings from our focus group interviews indicated that family caregivers found our study beneficial beyond the respite. The family caregivers reported that improved attitudes, physical function, and confidence in older adults with mild-to-moderate AD made it easier for them to provide care (Yu & Swartwood, 2012). Future studies could further explore the mechanisms for reduced caregiver distress from aerobic exercise interventions.

Two unique strengths of our study are the use of community setting to engage older adults with AD in aerobic exercise and the prescription and delivery of the intervention. Even for older adults without AD, there are limited community-based exercise programs and substantial gaps exist in community exercise facilities (Chard & Stuart, 2012). Those problems are accentuated for older adults with AD who likely require supervised exercise given their impaired judgment, decision-making, and cognition. Currently, most exercise studies in AD have been conducted in nursing home settings with not well-characterized AD samples (Forbes et al., 2008; Heyn, 2003; Yu, 2011). This study was able to enroll a well-characterized AD sample (confirmed AD diagnosis and staging using MMSE and CDR) and successfully delivered the intervention in community-based exercise settings. Older adults with mild-to-moderate AD achieved a 79% retention rate at 6 months with high adherence rate.

In addition, in our previous studies, we have developed a method for individualizing moderate-intensity aerobic exercise for older adults with AD (Yu et al., 2011). However, the heart rate reserve method had limited value for older adults with mild-to-moderate AD who had cardiac arrhythmia or were taking heart-rate-altering medications because their heart rate reserve could not be reliably determined. Our previous work showed that a combined heart rate and subjectively-reported exertion method produced a cardiac conditioning effect in older adults with mild-to-moderate AD (Yu, Savik, et al., 2011). Other strengths of our study included identified multiple strategies for successfully engaging older adults with AD in aerobic exercise, including establishment of rapport among staff, older adults with mild-to-moderate AD and family members, documenting exercise progress via an individual exercise log, which the older adults with mild-to-moderate AD used to share with families their progress, regular communications over the phone, and via the exercise log where the exercise interventionist documented the achievement for each older adult with mild-to-moderate AD for each exercise session, concerns, and reminders, providing transportation assistance, being flexible in scheduling, and providing reimbursement for gym memberships if membership costs were incurred.

The limitations of our pilot study are its small sample size and lack of a concurrent control group and comparisons with historical controls that must be interpreted with caution. The sample size of our study is comparable with other emerging exercise studies in AD and targeted the less-studied, community-based population (Arkin, 2003; Kemoun et al., 2010; Rolland et al., 2007; Williams & Tappen, 2007; Williams & Tappen, 2008). The decision to omit a control group was based on resource limitation, the purpose of refining the aerobic exercise intervention, and the availability of good natural history data. Providing supervised exercise intervention and transportation for participants were labor-intensive. The decision of not using a control group was also influenced by the anticipated recruitment difficulty: The recruitment rate to AD drug RCTs, defined as the number of older adults with mild-to-moderate AD qualified per site per month, ranged from 0.26 to 1.82 (Grill & Karlawish, 2010). A lot of resources were devoted to recruitment, so we were able to achieve 1.87 recruitment rate in this study. In addition, we were unsure whether it is feasible to engage older adults with AD in aerobic exercise with reasonable retention and compliance rates. Last, the available data from AD drug RCTs about the natural ADAS-Cog increase of 2.4 to 3.9 points over 6 months provided a good historical control to gauge our exercise effect on cognition (Doraiswamy et al., 2001). Despite its advantages, historical control has its inherent limitations, making it essential to interpret our results with caution. Because we have established the feasibility, retention, and compliance in this study, RCTs will be the natural next step to further evaluate the effects of aerobic exercise on AD symptoms.

Our study provides clinicians a practical way of prescribing and engaging community-dwelling older adults with AD in regular aerobic exercise because exercise has many established health benefits for older adults whether they slow AD progression or not.

## Conclusion

Our findings suggest that aerobic exercise may play a role in reducing declines in cognition, ADLs, and BPSD in older adults with AD. A community-program of aerobic exercise could significantly reduce caregiver distress.

## Figures and Tables

**Table 1:** Baseline Characteristics of Participants and Their Primary Family Caregivers.

	Older adults with mild-to-moderate AD, *N* = 26	Primary family caregivers, *N* = 26
Age (years)	78 ± 8 (60-91)	64 ± 12 (39-89)
Female (%)	62%	80%
BMI	27 ± 4 (19-40)	—
BMI category	Normal weight: 19% Overweight: 73% Obese: 8%	—
Non-Hispanic White	100%	100%
Years of education	16 ± 3 (12-24)	17 ± 3 (12-23)
MMSE	20 ± 4 (12-27)	—
MMSE category	MMSE 12-18: 27 % MMSE 19-27: 73 %	—
CDR	score = 1: 81% score = 2: 19%	—

*Note.* BMI = body mass index; normal weight BMI = 18.5-24.9; overweight BMI = 25-29.9; obese BMI > 30. MMSE = mini-mental state examination with higher score indicating better global cognition. CDR = clinical dementia rating score of 1 to 3 was required for eligibility; higher scores indicate more severe dementia.

* Values are mean ± standard deviation (range = minimum-maximum) or percent.

**Table 2:** Outcomes at Baseline and Changes From Baseline (mean ± *SE*) for 26 Older Adults With Mild-to-Moderate AD and Their Caregivers.

	Baseline	Month 3—Baseline	Month 6—Baseline
ADAS-Cog	18.2 ± 1	−0.6 ± 1.4	+1.9 ± 1.5
DAD (ADL)	25.7 ± 2	−0.4 ± 1	−1.1 ± 1
NPI-Q Severity (BPSD)	4.2 ± 0.8	−0.1 ± 0.6	−1.1 ± 0.7
NPI-Q Distress (Caregiver Distress)	5.3 ± 1	−0.4 ± 0.9	−2.1^a^ ± 1

ADAS-Cog = Alzheimer's Disease Assessment Scale–Cognitive Subscale, score 0 to 70; higher score indicates worse global cognition. DAD = Disability in Alzheimer's Disease, score 0 to 40; higher scores indicate greater disability. NPI-Q Severity = Neuropsychiatric Inventory– caregiver symptom severity, score 0 to 36; higher scores indicate more severe behavioral and psychological symptoms of dementia. NPI-Q Distress = score 0 to 60; higher scores indicate more distress due to behavioral and psychological symptoms of dementia.

a Significant change from baseline (*p* < .05).
